# Supplementary material for: How does abstract and concrete garbage classification signage influence waste sorting behavior?
Source: PeerJ. 2023 Dec 5;11:e16597. doi: 10.7717/peerj.16597 (PMC10704987; doi:10.7717/peerj.16597)
Supplement: Supplemental Information 1 [file peerj-11-16597-s001.docx]

Appendix Analysis of gender, educational level, and major under different conditions in Experiments 1 and 2.

|  | Gender | | | | Education level | | | | Major | | | |
| --- | --- | --- | --- | --- | --- | --- | --- | --- | --- | --- | --- | --- |
|  | Male | Female | t | p | Undergraduate | Postgraduate | t | p | Liberal arts | Science | t | p |
| Experiment 1 Abstract classification signage | | | | | | | | | | | | |
| food | 540.71±107.25 | 538.46±97.37 | 0.06 | 0.95 | 562.57±118.39 | 505.28±55.52 | 1.56 | 0.13 | 527.88±106.83 | 560±91.22 | -0.84 | 0.41 |
| batteries | 576.72±100.45 | 568.92±116.28 | 0.20 | 0.85 | 592.53±118.37 | 543.9±81.19 | 1.24 | 0.23 | 569.28±103.75 | 579.64±115.43 | -0.25 | 0.80 |
| chemicals | 576.98±98.76 | 592.19±126.4 | -0.37 | 0.71 | 606.92±128.84 | 549.81±67.45 | 1.41 | 0.17 | 585.91±110.59 | 580.91±116.37 | 0.12 | 0.91 |
| tubes | 605.54±114.77 | 609.36±117.43 | -0.09 | 0.93 | 613.05±134.2 | 598.74±79.38 | 0.33 | 0.74 | 608.11±122.14 | 605.97±104.12 | 0.05 | 0.96 |
| glass | 585.21±92.52 | 595.89±117.09 | -0.28 | 0.78 | 604.69±118.57 | 568.44±73.24 | 1.03 | 0.31 | 588.93±102.65 | 592.36±108.5 | -0.09 | 0.93 |
| metal | 597.84±104.5 | 617.3±125.33 | -0.46 | 0.65 | 630.3±127.84 | 571.86±78.87 | 1.41 | 0.17 | 601.16±115.53 | 616.87±113.52 | -0.36 | 0.72 |
| paper | 550.81±109.09 | 545.32±100.68 | 0.14 | 0.89 | 565.2±119.85 | 522.82±69.66 | 1.10 | 0.28 | 547.63±111.82 | 549.31±92.42 | -0.04 | 0.97 |
| plastic | 574.71±111.4 | 565.39±111.5 | 0.23 | 0.82 | 585.16±135.23 | 548.17±50.18 | 1.06 | 0.30 | 564.24±117.18 | 580.94±99.65 | -0.40 | 0.70 |
| textiles | 572.39±109.87 | 575.69±104.53 | -0.08 | 0.93 | 588.78±121.41 | 551.65±75.48 | 0.94 | 0.35 | 579.28±111.75 | 564.69±98.45 | 0.36 | 0.72 |
| Experiment 1 Concrete classification signage | | | | | | | | | | | | |
| food | 536.71±114.54 | 509.72±74.64 | 0.75 | 0.46 | 545.21±108.96 | 492.46±69.26 | 1.48 | 0.15 | 517.9±106.74 | 534.85±82.2 | -0.45 | 0.65 |
| batteries | 497.52±85.82 | 488.36±82.17 | 0.30 | 0.77 | 514.64±89.55 | 461.16±61.75 | 1.80 | 0.08 | 492.1±91.88 | 495.23±68.45 | -0.10 | 0.92 |
| chemicals | 545.86±94.76 | 535.98±60.72 | 0.34 | 0.74 | 556.18±79.27 | 518.86±77.75 | 1.27 | 0.21 | 546.87±91.29 | 531.54±56.3 | 0.50 | 0.62 |
| tubes | 519.11±99.59 | 502.42±73.8 | 0.52 | 0.61 | 534.75±96.92 | 476.17±58.2 | 1.87 | 0.07 | 506.77±100.01 | 519.17±63.7 | -0.37 | 0.72 |
| glass | 523.41±98.24 | 519.19±96.88 | 0.12 | 0.91 | 547.55±101.92 | 482.28±73.62 | 1.91 | 0.07 | 522.46±112.77 | 519.69±61.61 | 0.08 | 0.94 |
| metal | 519.66±91.92 | 507.57±76.37 | 0.39 | 0.70 | 531.23±91.77 | 488.2±65.48 | 1.40 | 0.17 | 516.17±91.95 | 510.31±71.58 | 0.18 | 0.86 |
| paper | 505.55±104.1 | 478.53±53.47 | 0.87 | 0.39 | 510.09±91.65 | 467.21±66.85 | 1.39 | 0.18 | 496.39±97.24 | 486.97±58.62 | 0.29 | 0.77 |
| plastic | 564.58±78.05 | 555.5±114.57 | 0.26 | 0.80 | 578.55±105.02 | 533.03±73.99 | 1.30 | 0.21 | 552.14±110.85 | 574.52±61.62 | -0.61 | 0.54 |
| textiles | 511.23±99.38 | 477.67±68.24 | 1.06 | 0.30 | 517.49±91.48 | 462.7±69.54 | 1.76 | 0.09 | 494.44±102.1 | 497.52±54.19 | -0.09 | 0.93 |
| Experiment 2 Traditional condition | | | | | | | | | | | | |
| food | 1012.43± 90.73 | 1033.23±143.53 | -0.49 | 0.63 | 1009.35±123.3 | 1039.58±103.15 | -0.70 | 0.49 | 1017.05±133.09 | 1027.19±90.33 | -0.24 | 0.82 |
| hazardous | 1083.31±104.82 | 1076.05±114.99 | 0.18 | 0.86 | 1055.24±95.67 | 1117.55±117.27 | -1.60 | 0.12 | 1091.96±102.35 | 1064.74±116.12 | 0.68 | 0.50 |
| recyclable | 1043.25± 97.26 | 1018.94±104.44 | 0.66 | 0.52 | 1017.71±92.23 | 1055.23±109.49 | -1.01 | 0.32 | 1044.33±95.04 | 1017.53±106.77 | 0.73 | 0.47 |
| residual | 1099.69± 99.74 | 1064.61±92.09 | 0.99 | 0.33 | 1073.75±93.89 | 1100.6±102.23 | -0.74 | 0.47 | 1093.72±84.08 | 1072.43±113.13 | 0.59 | 0.56 |
| Experiment 2 Nudging condition | | | | | | | | | | | | |
| food | 1000.66±80.75 | 1008.26±117.88 | -0.21 | 0.84 | 987.8±100.82 | 1028.18±88.91 | -1.13 | 0.27 | 1016.48±120.11 | 987.57±53.63 | 0.88 | 0.39 |
| hazardous | 1037.59±86.23 | 1057.59±87.41 | -0.63 | 0.54 | 1029.44±77.23 | 1071.49±95.08 | -1.33 | 0.19 | 1056.63±85.2 | 1032.7±88.14 | 0.75 | 0.46 |
| recyclable | 1025.62±90.59 | 1000.71±76.12 | 0.80 | 0.43 | 1000.03±71.53 | 1037.01±99.36 | -1.19 | 0.25 | 1029.17±87.43 | 996.06±79.07 | 1.07 | 0.29 |
| residual | 1042.46±92.51 | 1023.89±75.65 | 0.59 | 0.56 | 1019.38±81.11 | 1056.97±88.48 | -1.20 | 0.24 | 1043.15±78.6 | 1022.99±94.13 | 0.64 | 0.53 |
